# Childhood deaths from external causes in Estonia, 2001–2005

**DOI:** 10.1186/1471-2458-7-158

**Published:** 2007-07-17

**Authors:** Marika Väli, Katrin Lang, Ruth Soonets, Marika Talumäe, Andrej M Grjibovski

**Affiliations:** 1 Institute of Pathological Anatomy and Forensic Medicine, University of Tartu, 19 Ravila St, 50411 Tartu, Estonia; 2 Estonian Bureau of Forensic Medicine, Tallinn, Estonia; 3 Department of Public Health, University of Tartu, Estonia. 19 Ravila St, 50411 Tartu, Estonia; 4 Tartu University Children's Clinic, Tartu, Estonia. 6 Lunini St, 50411 Tartu, Estonia; 5 Tartu Support Centre for Abused Children, Estonia; 6 Division of Epidemiology, Norwegian Institute of Public Health, Postbox 4404 Nydalen, 0403 Oslo, Norway

## Abstract

**Background:**

In 2000, the overall rate of injury deaths in children aged 0–14 was 28.7 per 100000 in Estonia, which is more than 5 times higher than the corresponding rate in neighbouring Finland. This paper describes childhood injury mortality in Estonia by cause and age groups, and validates registration of these deaths in the Statistical Office of Estonia against the autopsy data.

**Methods:**

The data on causes of all child deaths in Estonia in 2001–2005 were abstracted from the autopsy protocols at the Estonian Bureau of Forensic Medicine. Average annual mortality rates per 100,000 were calculated. Coverage (proportion of the reported injury deaths from the total number of injury deaths) and accuracy (proportion of correctly classified injury deaths) of the registration of causes of death in Statistical Office of Estonia were assessed by comparing the Statistical Office of Estonia data with the data from Estonian Bureau of Forensic Medicine.

**Results:**

Average annual mortality from external causes in 0–14 years-old children in Estonia was 19.1 per 100,000. Asphyxia and transport accidents were the major killers followed by poisoning and suicides. Relative contribution of these causes varied greatly between age groups. Intent of death was unknown for more than 10% of injury deaths. Coverage and accuracy of registration of injury deaths by Statistical Office of Estonia were 91.5% and 95.3%, respectively.

**Conclusion:**

Childhood mortality from injuries in Estonia is among the highest in the EU. The number of injury deaths in Statistical Office of Estonia is slightly underestimated mostly due to misclassification for deaths from diseases. Accuracy of the Statistical Office of Estonia data was high with some underestimation of intentional deaths. Moreover, high proportion of death with unknown intent suggests underestimation of intentional deaths.

Reduction of injury deaths should be given a high priority in Estonia. More information on circumstances around death is needed to enable establishing the intent of death.

## Background

The United Nations Convention on the Right of the Child states that the child has the right to the highest attainable level of health and the right to a safe environment [1]. Moreover, mortality in infancy and childhood serve as good indicators of the population wellbeing. Injury is the most common cause of death among children and adolescents in Europe and is a major public health problem worldwide [2]. In spite of the fact that Europe enjoys the lowest average rates of childhood mortality in the world the rates vary considerably between European countries. Deaths from external cases account for an important fraction of the gap in overall childhood mortality between Eastern and Western Europe [3,4].

Estonia is a re-emerging country in North-eastern Europe, which regained its independence in 1991 after the collapse of the Soviet Union. In 2004, Estonia became a full member of the European Union. The total population of Estonia is 1,344,684 of which 202,429 (15.1%) are children from 0 to 14 years old (January 1, 2006).

**Figure 1:**
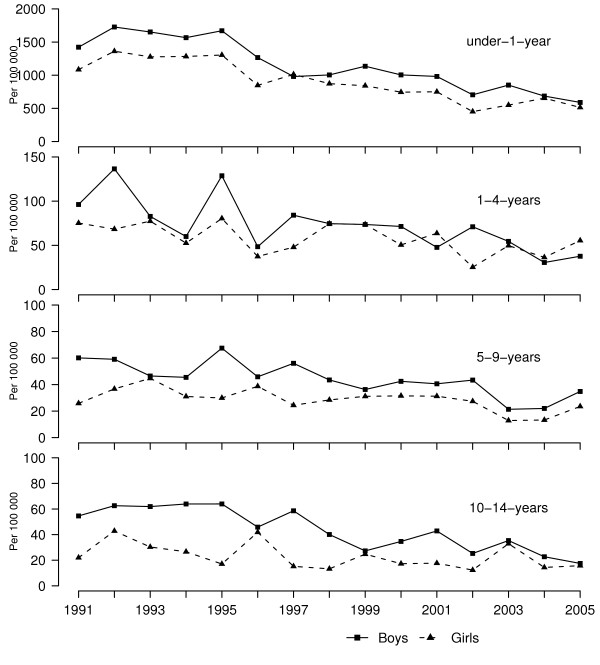
Mortality rates in children by age groups.

During the 15-year period of transition, infant mortality in Estonia decreased from 16.9 per 1000 live births in 1991 to 5.4 in 2005 reflecting considerable improvements in the country's economy and population's well-being. Less significant progress, however, has been observed for childhood and adolescent deaths (Figure 1), which may in part be explained by persistently high rates of injury mortality in these groups. In 2000, the overall rate of injury deaths in children aged 0–14 was 28.7 per 100000 in Estonia, which is more than 5 times higher than the corresponding rate in neighbouring Finland [5].

All injury deaths are preventable; therefore, detailed information on the occurrence and circumstances surrounding particular types of injury needs to be collected and analysed if effective interventions are to be developed [4]. The quality of data is an important prerequisite for the analysis of mortality patterns. However, to our knowledge, no analysis on either childhood mortality in Estonia or validity of the official death statistics in the country has been published.

This paper presents detailed description of injury deaths in 0–14-year-old children in Estonia in 2001–2005. Taking into account the concerns regarding domestic violence, ill treatment and abuse of children in families, at schools and in institutions in Estonia recently expressed by the UN Committee on the Rights of the Child [6], special attention was paid to the intent of death. In addition, we studied the validity of the data on external causes of death reported by the Statistical Office of Estonia by comparing the official data with the autopsy records.

## Methods

Data on the underlying cause of death were abstracted from the protocols of all medico-legal autopsies in children aged 0–14 years in Estonia from 2001 through 2005. The protocols were obtained from the Estonian Bureau of Forensic Medicine.

External causes of death were coded as V01-Y89 ("External causes of morbidity and mortality") as specified by ICD-10 [7]. According to the Estonian legislation, all deaths from external causes and cases of sudden or suspicious death are autopsied at Estonian Bureau of Forensic Medicine [8].

Information from the autopsy protocols was transferred to a registration form that included name, sex, dates of birth and death, cause of death and intent of death. By intent, injury deaths were classified as unintentional (accidents), intentional (suicides and homicides), and deaths with undetermined manner of death [9]. Circumstances around death were analysed using police reports that accompanied the protocols, and, where existing, verdicts of the court of law were also analysed.

The data were computerized and the part containing the data required for identification of cases was sent to Statistical Office of Estonia, which traced the underlying cause of death as registered in their database. In all cases of discrepancies in the cause of death between Estonian Bureau of Forensic Medicine and Statistical Office of Estonia as well as in cases where undetermined manner of death had been recorded at Estonian Bureau of Forensic Medicine, a thorough analysis of all accompanying documents was performed to determine the most probable intent of death. The data were analysed separately in four age groups: <1, 1–4, 5–9 and 10–14 year-olds.

Regarding other deaths registered at Statistical Office of Estonia, the cause of death was ascertained by either a pathologist who performed autopsy or a physician who examined the dead body and reviewed the medical records [10]. All coding of causes of death is performed at Statistical Office of Estonia using ICD-10 and following the adapted WHO guidelines for coding of the causes of death [11].

Given a short period of observation and relatively low number of cases in several categories, the mortality indices were calculated as average annual rates per 100,000 for the whole period from 2001 through 2005. The annual midyear population data for each age group were obtained from Statistical Office of Estonia.

In the validity study, the autopsy data were used as a "gold standard". The coverage (proportion of the reported injury deaths from the total number of injury deaths) and accuracy (proportion of correctly classified injury deaths) were calculated as percentages. Kappa statistics (κ) was calculated to adjust for chance agreement.

The study was approved by the Ethics Review Committee on Human Research at the University of Tartu (protocol no 101/2).

## Results

In 2001–2005, Estonian Bureau of Forensic Medicine performed 262 medico-legal autopsies of 0–14-year-old children, 40% (n = 105) of which were girls and 60% (n = 157) boys. Altogether, 211 of deaths in the autopsied children (81%) were attributed to the external causes. Thus, the average annual mortality from injuries in Estonia was 19.1 per 100,000. The remaining 51 cases were confirmed to be caused by diseases and are not discussed here.

Unintentional deaths constituted 80% of deaths from external causes with asphyxia and transport accidents being the main killers followed by poisonings and other unintentional injuries (Table 1).

**Table 1:** Age-specific average annual mortality from external causes in 0–14-year-old Estonian children, 2001–2005.

Cause of death	Age									
		
	<1 year		1–4 years		5–9 years		10–14 years		Total	
	N	per 100000 (95% CI)	N	per 100000 (95% CI)	N	per 100000 (95% CI)	N	per 100000 (95% CI)	N	per 100000 (95% CI)

Unintentional injuries	37	56.1 (40.8–77.3)	47	18.8 (14.1–25.0)	41	12.6 (9.3–17.0)	44	9.5 (7.1–12.8)	169	15.3 (13.2–17.8)
Asphyxia	34	51.6 (37.0–72.0)	24	9.6 (6.5–14.3)	13	4.0 (2.3–6.8)	12	2.6 (1.5–4.5)	83	7.5 (6.1–9.3)
Drowning	1	1.5 (0.4–8.4)	13	5.2 (3.1–8.9)	13	4.0 (2.3–6.8)	6	1.3 (0.6–2.8)	33	3.0 (2.1–4.2)
Aspiration	26	39.4 (27.0–57.7)	5	2.0 (0.9–4.7)	0		2	0.4 (0.1–1.6)	33	3.0 (2.1–4.2)
Strangulation	1	1.5 (0.4–8.4)	2	0.8 (0.2–2.9)	0		2	0.4 (0.1–1.6)	5	0.5 (0.2–1.1)
Other	6	9.1 (4.3–19.8)	4	1.6 (0.6–4.1)	0		2	0.4 (0.1–1.6)	12	1.1 (0.6–1.9)
Transport accidents	2	3.0 (0.9–10.9)	13	5.2 (3.1–8.9)	17	5.2 (3.3–8.3)	25	5.4 (3.7–8.0)	57	5.2 (4.0–6.7)
Poisoning	1	1.5 (0.4–8.4)	7	2.8 (1.4–5.8)	5	1.5 (0.7–3.6)	3	0.6 (0.2–1.9)	16	1.4 (0.9–2.4)
Electric trauma	0		0	0	2	0.6 (0.2–2.2)	1	0.2 (0.0–1.2)	3	0.3 (0.0–0.8)
Hypothermia	0		1	0.4 (0.0–2.2)	0		0		1	0.1 (0.0–0.5)
Falls	0		2	0.8 (0.2–2.9)	2	0.6 (0.2–2.2)	1	0.2 (0.0–1.2)	5	0.5 (0.2–1.1)
Hit by object	0		0		2	0.6 (0.2–2.2)	2	0.4 (0.1–1.6)	4	0.4 (0.1–0.9)
Intentional injuries	5	7.6 (3.3–17.7)	0		2	0.6 (0.2–2.2)	13	2.8 (1.7–4.8)	20	1.8 (1.2–2.8)
Suicides	0		0		1	0.3 (0.0–1.7)	12	2.6 (1.5–4.5)	13	1.2 (0.7–2.0)
Homicides	5	7.6 (3.3–17.7)	0		1	0.3 (0.0–1.7)	1	0.2 (0.0–1.2)	7	0.6 (0.3–1.3)
Undetermined manner of death	16	24.3 (15.0–39.4)	4	1.6 (0.6–4.1)	0		2	0.4 (0.1–1.6)	22	2.0 (1.3–3.0)
Total	58	88.1 (68.1–113.6)	51	20.4 (15.5–26.8)	43	13.2 (9.8–17.7)	59	12.8 (9.9–16.5)	211	19.1 (16.7–21.9)

Asphyxia was a cause of almost half of all unintentional deaths in 2001–2005. Aspiration was registered as the most frequent cause of asphyxia in infants while in other age groups its contribution was small. Strangulation was registered as a cause of death in 5 cases: 2 children accidentally hung themselves while playing, 2 died strangulated by hood (one on the fence post, another on the barbed-wire fence) and 1 child was caught by pants by the cot bar. Among the 12 deaths classified as "other asphyxia", there were 4 accidental obstructions of external airways, 4 cases of choking and 4 thoracic immobilizations (one child was trapped under the fallen cupboard, one was caught between the bed and the wall, one was caught under the cement box, and one between the pram parts).

Drowning was the leading cause of death among the cases of asphyxia in all age groups except infants. It was the most frequent cause of death in 1–4-year-old children together with transport accidents, second in 5–9 year-olds and third in 10–14 year-olds. Most of the children drowned in outdoor waters.

Transport accidents were the leading cause of death with similar rates of 5.2–5.4 per 100,000 children in all age groups except infants. Road traffic accidents were the cause of death in 47 of 57 deaths in transport accidents. Nineteen children were passengers in the motor vehicles, 7 were pedestrians and 21 died in bike accidents. Deaths from transport accidents which could not be classified as road traffic accidents included 4 deaths in railway accidents, one in airplane, one in motorbike, and one in snowmobile accidents. Two children were overrun by cars outside the roads (one in a tent and one in a pram).

Among the 16 deaths from poisoning, 10 were caused by carbon monoxide while the remaining 6 were the cases of poisonings with drugs. Three deaths were caused by electric trauma and there was one case of hypothermia (one infant was found dead in the yard). Among "other" deaths from injuries (n = 9), there were 5 falls from heights (4 from windows and one from a tractor) and 4 children were hit by a falling object.

Altogether, 20 deaths were classified as intentional deaths. Ten boys and three girls committed suicides (all by hanging). Seven deaths were classified as homicide. In one case, the father killed his whole family, including the 6-year-old son; in another case, the mother's partner killed her 11-year old son. All other cases were infanticide. Strangulation was the cause of 2 of these deaths while the rest of the cases were characterized by traumas of the skull including one case of the shaken baby syndrome.

For the remaining 22 deaths (10.4% of all injury deaths) the manner of death was undetermined. These cases included 6 unclear traumas of the skull, 5 unclear mechanic asphyxications, 7 bodies of newborn babies were found and in 4 cases the bodies were found in advanced stages of putrefaction so that the underlying cause of death and intent were impossible to identify. The rationale for classifying these deaths as of undetermined manner of death was that death from a disease had been excluded at the autopsy and/or the evidence from police suggested that the death was unnatural.

Eight autopsied children (3.1%) could not be linked to the corresponding records in Statistical Office of Estonia implying that deaths and/or births were not registered. Two of them were the cases of death from diseases (one child died in Finland and the death of another child was registered with a considerable delay). The other six unregistered cases were the dead bodies found and registered as late foetal deaths.

The total coverage of the deaths from external causes by Statistical Office of Estonia was 91.5%. Exclusion of cases that could not be linked to Statistical Office of Estonia increases the coverage to 94.1%. The underestimation of deaths from external causes by Statistical Office of Estonia is explained by misclassification of 4 unintentional injuries, 7 injuries with undetermined manner of death and one infanticide as deaths from diseases.

The overall accuracy of registration of deaths from external causes by Statistical Office of Estonia was 95.3% if the deaths were dichotomised either as deaths from external causes or from diseases. Adjustment for agreement by chance showed almost perfect agreement (κ = 0.80).

One unintentional injury with asphyxia as a cause of death was misclassified by Statistical Office of Estonia as suicide. Eight cases were classified as deaths from unintentional injuries by Statistical Office of Estonia while the Estonian Bureau of Forensic Medicine could not exclude intentional deaths and thus classified them as deaths with undetermined manner of death. These included 6 cases of asphyxia, a shooting injury and a fatal burn. There was not enough evidence to confirm one case of homicide registered in Statistical Office of Estonia and thus this case was classified as death with undetermined manner.

Given that the forensic experts could not define the manner of death for the 9 cases of deaths from external causes both the best and the worst case scenarios were considered for the accuracy calculations in relation to deaths from external causes. Under assumption that all 9 cases were correctly classified by Statistical Office of Estonia, the accuracy would be 94.9% (κ = 0.90). If all these cases were assumed to be misclassified, the accuracy would decrease to 91.3% (κ = 0.83).

## Discussion

This first nation-wide autopsy-based study on causes of death in 0–14 years old Estonian children revealed that the average annual level of mortality of external causes was high and the most common causes of death were asphyxia and transport accidents, though the relative contribution of the causes varies between the age-groups. Moreover, we observed that injury deaths were slightly underestimated in Statistical Office of Estonia due to registering deaths from external causes as deaths from diseases. We also observed that 95% of all registered at Statistical Office of Estonia injury deaths were classified correctly according to the autopsy results.

The overall injury mortality in 2001–2005 was 19.1 per 100,000 children. This is almost 5 times higher than in Finland [12,13], 3 times higher than in Canada [14] during the same period, and is comparable with the rates in these countries in mid-1970s and in early 1980s, respectively.

The reduction in childhoodmortality from external causesfrom 28.7 per 100 000 in 2000 to the observed level indicates that some progress in reducing injury mortality in children in Estonia has been achieved over the recent years. Reasons for this kind of improvement could be attributed, along with general improvements in the country, to the National Health Programme for Children and Youth, approved by the Estonian Government in the year 2000. Among the aims of the programme was "reduce injuries caused by external origins and traumas and increase safety of the environment". It has been acknowledged that the health of children and youth improved as a result of understanding that "the key to improving the health of children and the entire population lies not only within the public health system and the Ministry of Social Affairs but also within many other fields and ministry administrative domains and in accompanying activities" [15].

The most pronounced differences in deaths from injuries between Estonia and other countries are in infants. Estonian infant mortality due to injuries is currently higher than it was 30 years ago in Finland. The Estonian rates are only slightly lower than those in Russia (99.2 per 100,000), but considerably higher than the rates in the USA (19.7), France (14.3), Japan (23.3) or Canada (15.0) [14,16]. More than half of all injury deaths in infants were due to asphyxia (mainly aspiration), which is in agreement with the findings from the USA [14] and the UK [9]. However, the corresponding rate was 2.7 per 100 000 in the latter study, which is about twenty times lower than in Estonia. The relatively high rate of asphyxia deaths may have occurred due to the routine forensic practice in Estonian not recording the underlying causes of death (leading to asphyxia) in these cases, but rather the direct cause of death (asphyxia). However, for this study we used data as recorded in the forensic autopsy protocols.

The most worrying observation is that in 8.6% of all infant deaths were due to homicide. The information on intentional deaths in infancy is scarce. However, several authors emphasize that infants are at increased risk of being battered to death [17,18]. Another concern is that in case of further 27.6% of infant deaths the manner of death was undetermined which raises suspicion about considerable underestimation of infanticide in Estonia.

Since the difference between injury mortality compared to Estonia and other countries is largest in infants, some of the reasons could be given. The evidence is very difficult to establish because of lack of information, but our continuing research has shown (paper in preparation) that harmful habits such as alcohol and/or drug abuse, leading to child neglect, are rather frequent reasons leading to infant injury deaths.

Regarding the distribution of external causes in all age groups above 1 year of age, asphyxia is rather frequent, but declining with age with drowning becoming the main cause of asphyxia in the older age. Transport accidents with a similar rate of about 5 per 100,000 children are also rather frequent. Pan et al. reported that traffic accidents were the most common cause of injury deaths among children aged 1–14 in Canada, 1979–2002, followed by suffocation and drowning [14] that corresponds to our findings. These are preventable deaths and yet they form the single largest cause of death in European children [19].

McKee and Oreskovic [19] claim that child injuries are low at the policy agenda in Europe. The reasons given were: 1) child injuries are typically an invisible condition, 2) as a policy issue they have no owner, and 3) injuries are still seen as something that just happens. In Estonia, although some success in reducing child injury fatalities has been achieved, these three issues should be approached. This would include establishing injury databases and increasing research on injuries, as injury epidemiology itself is not an active part of mainstream epidemiology [20]. It should also include actions by a wide range of people, including politicians to develop the policies on safety, and strengthening public health services. Last but not least, child and especially infant injury prevention in Estonia should include education of parents and childminders, with special attention to families with low educational background and marginalized groups.

A high proportion of deaths with undetermined manner especially in infants is the major limitation of this study. The reason for that is the lack of information accompanying the case referred to by the medico-legal autopsy. Often the forensic pathologist does not attend the examination of the scene of death, and his/her conclusions about the intent of death are based only on the autopsy findings. Determining the intent of death is especially difficult in children under 1 year of age, which may lead to considerable underestimation of infant homicide. Brenner et al. [22] reported that in the United States, the major cause of injury deaths in infants was homicide, followed by suffocation, motor vehicle crashes, and choking. Several studies found substantial underestimation of homicides as a result of child abuse, including infants, in vital records systems [23,24]. Death certificates have been found to underestimate deaths related to child abuse by about 50% [25]. In the USA, child homicide victims are generally young children dying from blunt head injury [26]. Such lethal injuries were described in our study, with manner of death undetermined, and it leads to the conclusion that these might, in fact, be homicides. More information is needed to be able to establish the intent of death in these cases. Thus, health professionals have a great responsibility in providing information regarding the circumstances of death, as they are often the first to see the (dead) child and the presumable crime scene. They have to record all available evidence that may help pathologist to decide whether the cause of death was natural or if there could be any suspicion to death from external cause. In suspicious cases they have to inform the police who carries out the crime scene investigation and orders forensic medical expertise. In several cases the bodies were found at an advanced stage of putrefaction, which made it impossible for the pathologist to determine the intent of death, though the intentional death might have been suspected.

On the other hand, we analysed all cases of autopsied children between 0 and 14 years in Estonia in 2001–2005, which is the major strength of the study.

We revealed that the coverage of external causes of death in Estonian children by Statistical Office of Estonia is high. The accuracy of the registration is also high. The kappa estimates suggest almost perfect agreement according to the guidelines provided by Landis and Koch [27] or very good agreement according to Altman [28] even in the worst case scenario. The fact that the majority of cases of discrepancy refer to cases where undetermined manner of death as classified by Estonian Bureau of Forensic Medicine was classified as an unintentional death or death from diseases by Statistical Office of Estonia, suggests underestimation of intentional deaths in official statistics.

## Conclusion

Childhood mortality from injuries in Estonia is among the highest in the EU. The quality of the registration of deaths from external causes by Statistical Office of Estonia is high, which implies that the data from Statistical Office of Estonia could be used in large-scale epidemiological research. However, one has to keep in mind that the number of injury deaths in Statistical Office of Estonia is slightly underestimated mostly due to misclassification as deaths from diseases and missing data on some births. Moreover, high proportion of deaths with undetermined manner suggests underestimation of intentional deaths especially in infants. More information on circumstances around death is needed to enable establishing the manner of death. Reduction of injury deaths should be included in the current public health agenda and given a high priority in Estonia.

## Competing interests

The author(s) declare that they have no competing interests.

## Authors' contributions

This study was devised by MV, KL and RS. MT organised and undertook the abstraction of autopsy protocols. KL arranged and carried out the linkage to the data of the Statistical Office of Estonia. KL and AG analysed the data. MV, KL and AG drafted the paper. The interpretation of the results and the final draft of the paper involved input from all five authors. All authors have read and approved the final manuscript.

**Table 2:** Validation of causes of death in all autopsied 0–14 years old children in 2001–2005.

Cause of death by Statistical Office of Estonia		Manner of death by forensic pathologist					
		
			External causes				
				
		Disease	Unintentional injury	Suicide	Homicide	Undetermined manner of death	Total
Disease (AA00-R99)		*49*	4	-	1	7	61
External (V01-Y99)	unintentional injury (V01-Y99, W00-X59)	-	*164*	-	-	8	172
	suicide(X60-X84)	-	1	*13*	-	-	14
	homicide(X85-Y09)		-		*6*	1	7
Registration missing		2	-	-	-	6	8
Total		51	169	13	7	22	262

## Pre-publication history

The pre-publication history for this paper can be accessed here:



## References

[B1] United Nations (1989). Convention on the Rights of the Child New York.

[B2] Crawley T (1996). Childhood injury: significance and prevention strategies. J Pediatr Nurs.

[B3] McKee M, Oreskovic S (2002). Childhood injury: call for action. Croat Med J.

[B4] (1998). Childhood injuries A priority area for the transition countries of Central and Eastern Europe and the Newly Independent States Final Report.

[B5] WHO Mortality databank (2003). Mortality indicators by 67 causes of death, age and sex (HFA-MDB), offline version.

[B6] (2004). UN Committee on the Rights of the Child Report.

[B7] World Health Organization (1992). International Statistical Classification of Diseases and Related Health Problems, tenth revision.

[B8] (2006). Surma põhjuse tuvastamise seadus Law of determination of Cause of death.

[B9] Roberts I, DiGuiseppi C, Ward H (1998). Childhood injuries: extent of the problem, epidemiological trends, and costs. Inj Prev.

[B10] Lang K (2000). Death certification, coding and registration in Estonia.

[B11] (1997). Juhend arstliku surmatunnistuse täitmiseks ja väljastamiseks Guidelines for compiling and issuing medical death certification.

[B12] Parkkari J, Kannus P, Niemi S, Koskinen S, Palvanen M, Vuori I, Järvinen M (2000). Childhood deaths and injuries in Finland in 1971–1995. Int J Epidemiol.

[B13] Mattila VM, Parkkari J, Niemi S, Kannus P (2005). Injury-related deaths among Finnish adolescents in 1971–2002. Injury.

[B14] Pan SY, Ugnat AM, Semenciw R, Desmeules M, Mao Y, Macleod M (2006). Trends in childhood injury mortality in Canada, 1979–2002. Inj Prev.

[B15] (2005). Laste ja noorukite terviseprogramm aastani 2005 National Health Programme for Children and Youth until 2005.

[B16] (2001). Demoscope weekly Electronic version of bulletin Naselenie i obschestvo (Population and Society).

[B17] McClain PW, Sacks JJ, Froehlke RG, Ewigman BG (1993). Estimates of fatal child abuse and neglect, United States, 1979 through 1988. Pediatrics.

[B18] Björnstig U, Björnstig J, Ahlm K, Sjögren H, Eriksson A (2006). Violent deaths in small children in northern Sweden. Int J Circumpolar Health.

[B19] Lyons RA, Brophy S (2005). The epidemiology of childhood mortality in European Union. Current Paediatrics.

[B20] McKee M, Oreškoviæ S (2002). Childhood injury: call for action. Croatian Medical Journal.

[B21] Petridou E (2000). Childhood injuries in the European Union: can epidemiology contribute to their control?. Acta Pædiatr.

[B22] Brenner RA, Overpeck MD, Trumble AC, DerSimonian R, Berendes H (1999). Deaths attributable to injuries in infants, United States, 1983–1991. Pediatrics.

[B23] Overpeck MD, Brenner RA, Trumble AC, Smith GS, MacDorman MF, Berendes HW (1999). Infant injury deaths with unknown intent: what else do we know?. Inj Prev.

[B24] Herman-Giddens ME, Brown G, Verbiest S, Carlson PJ, Hooten EG, Howell E, Butts JD (1999). Underascertainment of child abuse mortality in the United States. JAMA.

[B25] Crume TL, DiGuiseppi C, Byers T, Sirotnak AP, Garrett CJ (2002). Underascertainment of child maltreatment fatalities by death certificates, 1990–1998. Pediatrics.

[B26] King WK, Kiesel EL, Simon HK (2006). Child abuse fatalities: are we missing opportunities for intervention?. Pediatr Emerg Care.

[B27] Landis JR, Koch GG (1977). The measurement of observer agreement for categorical data. Biometrics.

[B28] Altman DG (1991). Practical statistics for medical research.

